# Pulp Revascularization in Immature Permanent Tooth with Apical Periodontitis Using Mineral Trioxide Aggregate

**DOI:** 10.1155/2014/564908

**Published:** 2014-05-14

**Authors:** Katsura Saeki, Yuko Fujita, Yasuhiro Shiono, Yasuhiro Morimoto, Kenshi Maki

**Affiliations:** ^1^Department of Pediatric Dentistry, Kyushu Dental University, 2-6-1 Manazuru, Kokurakita-ku, Kitakyushu 803-8580, Japan; ^2^Department of Oral Diagnostic Science, Kyushu Dental University, Kitakyushu 803-8580, Japan

## Abstract

Mineral trioxide aggregate (MTA) is a material that has been used worldwide in several clinical applications, such as apical barriers in teeth with immature apices, repair of root perforations, root-end filling, pulp capping, and pulpotomy. The purpose of this case report was to describe successful revascularization treatment of an immature mandibular right second premolar with apical periodontitis in a 9-year-old female patient. After preparing an access cavity without anesthesia, the tooth was isolated using a rubber dam and accessed. The canal was gently debrided using 5% sodium hypochlorite (NaOCl) and 3% hydrogen peroxide irrigant. And then MTA was packed into the canal. X-ray photographic examination showed the dentin bridge 5 months after the revascularization procedure. Thickening of the canal wall and complete apical closure were confirmed 10 months after the treatment. In this case, MTA showed clinical and radiographic success at revascularization treatment in immature permanent tooth. The successful outcome of this case suggests that MTA is reliable and effective for endodontic treatment in the pediatric dentistry.

## 1. Introduction

Immature permanent teeth with apical periodontitis or an abscess are generally treated by apexification [1]. However, revascularization procedures have recently been recommended to treat immature permanent teeth with necrotic pulp tissue and/or apical periodontitis or an abscess.

Mineral trioxide aggregate (MTA) is a material used worldwide in a variety of clinical applications, such as an apical barrier for teeth with immature apices, repair of root perforations, root-end filling, pulp capping, and pulpotomy procedures [2–9]. In this paper successful revascularization treatment of an immature mandibular right second premolar with apical periodontitis in a 9-year-old patient using MTA was described.

## 2. Case Report

**Figure 1:**
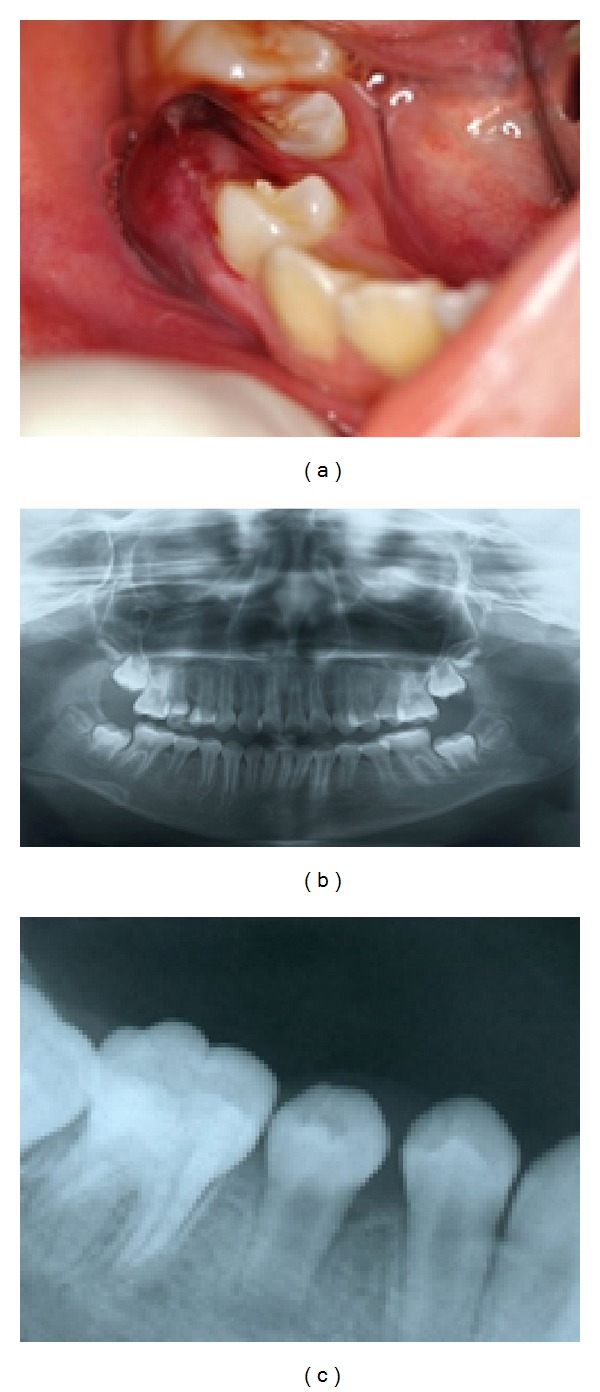
(a) Preoperative intraoral photograph showing a gingival abscess in the mandibular right second premolar. (b) Panoramic X-ray showing extensive radiolucency in the periradicular region in the mandibular right second premolar compared with the mandibular left second premolar. (c) X-ray showing an immature open apex and enlargement of the periodontal ligament space and extensive radiolucency in the periradicular region in the mandibular right second premolar.

In June 2012, a 9-year-old Japanese girl was referred to private clinic by a general dentist for detailed examination of a gingival abscess in the mandibular right second premolar. The medical history of the patient was unremarkable, and there was no relevant family history of medical or dental abnormalities. An extraoral examination revealed swelling in the buccal region and the patient complained of spontaneous pain. Furthermore, an intraoral examination revealed a gingival abscess in the region of the mandibular right second premolar (Figure 1(a)). The percussion test was positive.

Radiographic findings showed enlargement of the periodontal ligament space, along with extensive radiolucency in the periradicular region in the area of the mandibular right second premolar as compared with the mandibular left second premolar (Figures 1(b) and 1(c)). The pulp vitality test was negative. The clinical diagnosis was acute periradicular periodontitis of the mandibular right second premolar with pulpal necrosis.

**Figure 2:**
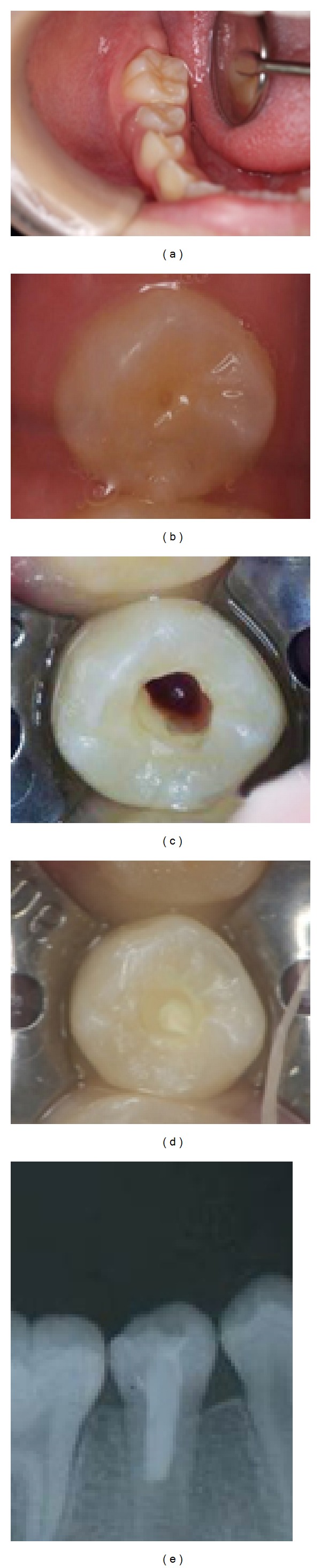
(a) Intraoral photograph showing no abnormalities of gingiva. (b) The central cusp of the mandibular right second premolar had been fractured. (c) After controlling hemorrhage, viable tissue was observed in the canal because insertion of a K-file evoked a sensation. (d) Placement of MTA in the canal. (e) Postoperative X-ray photograph showing MTA placement in canal.

The patient underwent oral surgery at Kyushu Dental University Hospital. The reason for hospitalization was because she was not able to eat for gingival swelling and spontaneous pain. During hospitalization, she received an intravenous drip containing an antibiotic. She left the hospital 6 days later and was referred to our clinic. Postsurgery intraoral examination showed no abnormalities on the gingiva of the mandibular right second premolar (Figure 2(a)). However, the talon cusp of the mandibular right second premolar was fractured (Figure 2(b)). Without using anesthetic, the tooth was isolated with a rubber dam and accessed. Upon entering the coronal aspect of the root canal, hemorrhaging into the pulp chamber was observed (Figure 2(c)). A file of 10 K size was inserted into the canal. The length of file is 10 mm. And the patient reported discomfort, indicating potential survival of residual vital pulp tissue. The hemorrhaging in the coronal portion of the canal was gently irrigated; then the area was debrided using 1.5 mL of 5% sodium hypochlorite (NaOCl) and 1.5 mL of 3% hydrogen peroxide [2, 10]. No instrumentation was performed. Next, MTA (Pro-Root MTA, Dentsply Sankin, Tochigi, Japan) was packed into the canal using MAP system (Dentsply Sankin, Tochigi, Japan) (Figure 2(d)) and the access cavity was closed with glass-ionomer cement (Fuji IX GP, GC, Tokyo, Japan). An X-ray obtained after the procedure confirmed MTA placement in the canal (Figure 2(e)).

**Figure 3:**
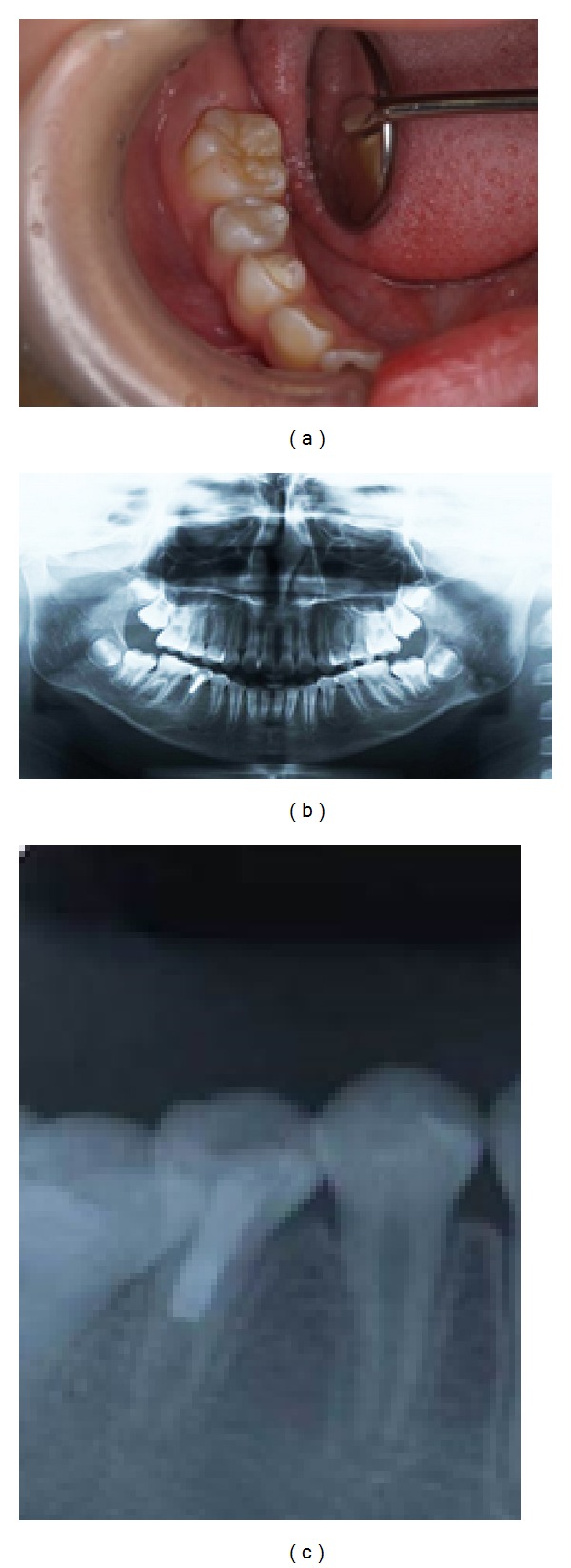
(a) Intraoral photograph showing no abnormalities of gingiva. (b) Panoramic X-ray photograph showing the formation of a dentin bridge in the mandibular right second premolar. (c) X-ray shows that radiolucency became less radiolucent in the periradicular region and the formation of a dentin bridge in the mandibular right second premolar.

**Figure 4:**
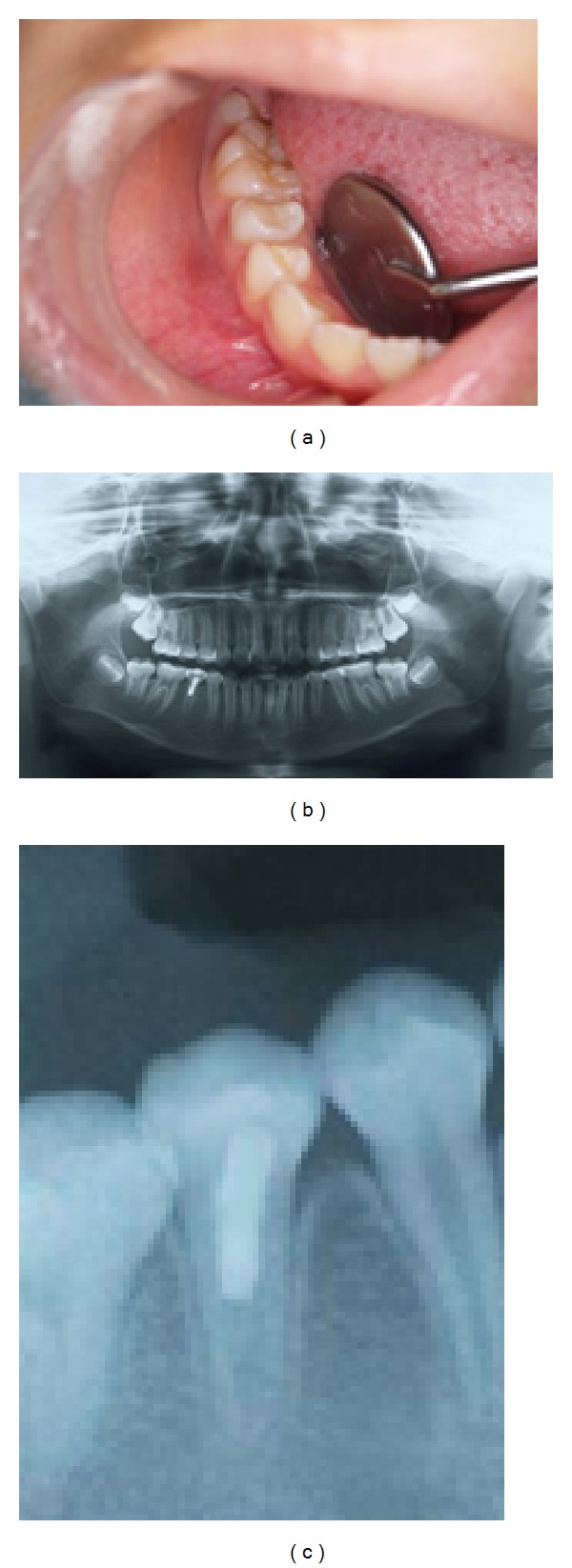
(a) Intraoral photograph showing no abnormalities of gingiva. (b) Panoramic X-ray showing the formation of a dentin bridge and thickening of the canal walls in the mandibular right second premolar. (c) Panoramic X-ray showing the formation of a dentin bridge and thickening of the canal walls and establishment of the periodontal ligament space and lamina dura in the mandibular right second premolar.

Six months later, an intraoral examination showed no abnormalities in the gingiva of the mandibular right second premolar (Figure 3(a)), while an X-ray photographic evaluation showed formation of a dentin bridge in the mandibular right second premolar (Figures 3(b) and 3(c)). Ten months later, an intraoral examination showed no abnormalities of the gingiva in the mandibular right second premolar (Figure 4(a)), and X-ray images revealed formation of a dentin bridge and thickening of the canal walls in the mandibular right second premolar (Figures 4(b) and 4(c)).

We think it is important to follow up this tooth, but she had moved. It is too far for her to refer to our clinic. So we could not follow up her.

## 3. Discussion

Apexogenesis is done is in immature teeth when part of the pulp tissue inside the root canal remains vital and apparently healthy. This procedure allows continued physiological development and formation of the root end.

In cases with an immature root with a large apical foramen, pulp infection associated with an apical lesion does not always indicate pulp necrosis, as seen in our patient, likely because the pulp at this stage is vital enough and has extremely high healing ability. Those procedures have been shown to result in increased thickening of the canal walls by deposition of hard tissue and encourage continued root development in affected immature permanent teeth [10–13]. Continued root development of revascularization of immature permanent necrotic teeth depends on whether Hertwig's epithelial sheath survives in cases of apical periodontitis/abscess.Hertwig's epithelial sheath has important role in root development and shape and may be involved in regulation of the differentiation of periodontal ligament stem cells with the formation of cementum.

MTA is a cement material with excellent biocompatibility and good sealing capacity that is able to produce hard tissues such as dentin and cementum [14]. It is used for apexification and sealing of communication between the root and periodontal tissue, such as in reverse root canal filling and perforation repair [15]. However, there are few reports of its use for pulp revascularization using MTA [11, 16]. Calcium hydroxide formulations are typically used for apexogenesis and poor sealing capacity [17]. Furthermore, formation of a necrotic layer immediately beneath the pulp can occur and the procedure must be changed to a pulpectomy in some cases due to spreading inflammation, as it does not provide an adequate biodefense mechanism against even a limited bacterial invasion [18]. Accordingly, we used MTA in this case. Generally, the root of a tooth with pulp revascularization is smaller than a mature tooth and is characterized by more rapid calcification of the pulp than that seen after a conventional apexogenesis procedure, as noted in the present case [11]. Chen et al. [11] demonstrated five types of response of these immature teeth with infected necrotic pulp tissue and apical periodontitis/abscess to revascularization procedures: type 5, formation of a hard tissue barrier in the canal between the coronal cement plug and root apex using MTA. The present case was consistent with type 5. In cases with an immature root with a large apical foramen, pulp infection associated with an apical lesion does not always indicate pulp necrosis, as seen in our patient, likely because the pulp at this stage is vital enough and has extremely high healing ability. It has been reported that pulp revascularization was induced by removing infective material from the root canal and applying calcium hydroxide past MTA [10, 12]. Traditionally, in the clinical protocol for revascularization treatment, several kinds of antibacterial medicine were used. But, recently, it reported that a single-visit pulp revascularization protocol can be a favorable treatment for partially necrotic immature permanent teeth using MTA as a pulpal seal [16].

Accordingly, for immature tooth with a pulp infection and open apical foramen, treatment should start with pulp revascularization and then shift to apexification if incurable, while considering the infection to be reversible.

## 4. Conclusion

In the present case, clinical and radiographic evidence showed successful use of MTA for revascularization treatment of an immature permanent tooth. More studies are necessary to understand the mechanisms of pulp revascularization comparing different protocols.
